# Unmatched Case-Control Study on Late Presentation of HIV Infection in Santiago, Cape Verde (2004–2011)

**DOI:** 10.3390/ijerph13030320

**Published:** 2016-03-15

**Authors:** António L. Moreira, Inês Fronteira, Gonçalo Figueiredo Augusto, Maria Rosario O. Martins

**Affiliations:** 1 Ministry of Health, Palácio do Governo, Várzea-Praia C.P. 47, Santiago-Cape Verde; antonio.moreira@ms.gov.cv; 2 Global Health and Tropical Medicine (GHTM), Institute of Hygiene and Tropical Medicine-NOVA University of Lisbon (IHMT-UNL), Rua da Junqueira 100, Lisbon 1349-008, Portugal; ifronteira@ihmt.unl.pt (I.F.); figueiredo.augusto@ihmt.unl.pt (G.F.A.)

**Keywords:** Cape Verde, HIV/AIDS, late presentation, CD4 count

## Abstract

Access to free antiretroviral therapy (ART) in Sub-Saharan Africa has been steadily increasing over the past decade. However, the success of large-scale ART programmes depends on timely diagnosis and early initiation of HIV care. This study characterizes late presenters to HIV care in Santiago (Cape Verde) between 2004 and 2011, and identifies factors associated with late presentation for care. We defined late presentation as persons presenting to HIV care with a CD4 count below 350 cells/mm^3^. An unmatched case-control study was conducted using socio-demographic and behavioural data of 368 individuals (191 cases and 177 controls) collected through an interviewer-administered questionnaire, comparing HIV patients late and early presented to care. Logistic regression was performed to estimate odds ratio and 95% confidence intervals. Results show that 51.9% were late presenters for HIV. No differences were found in gender distribution, marital status, or access to health services between cases and controls. Participants who undertook an HIV test by doctor indication were more likely to present late compared with those who tested for HIV by their own initiative. Also, individuals taking less time to initiate ART are more likely to present late. This study highlights the need to better understand reasons for late presentation to HIV care in Cape Verde. People in older age groups should be targeted in future approaches focused on late presenters to HIV care.

## 1. Introduction

Globally, 36.9 million people were living with HIV and 2 million became newly infected in 2014 [1]. With 25.8 million people living with HIV and 1.4 million new HIV infections, Sub-Saharan Africa remains the most affected region in the global AIDS epidemic [1]. Access to antiretroviral therapy (ART) has increased dramatically in this region. While in 2005 ART reached 810,100 people living with HIV, more than 10.7 million adults and children infected with HIV received lifesaving treatment in 2014 [1,2].

In Cape Verde 72% of HIV cases are caused by HIV-1, 22% are caused by HIV-2, and 6% by both infections [3,4]. The first ever isolation of HIV-2 was in an AIDS patient from Cape Verde who developed symptoms in 1982 [5]. Serological tests for HIV-1 and HIV-2 have been available in Cape Verde since 1987 [3].

Following the first detections of HIV in 1986, the accumulated number of HIV patients increased to 4049 by 2012, of which 1340 developed AIDS and 866 died from AIDS-related diseases [6]. HIV prevalence in Cape Verde in 2005 was 0.8%, being higher amongst men (1.1%) than amongst women (0.4%) [6]. Incidence of HIV has been rising in the last decade, having increased from 37.5 per 100,000 in 2003 to 69.4 per 100,000 in 2012 [6]. HIV prevalence in sex workers is estimated at 5.3% and in pregnant women tested at sentinel sites is less than 1% [4].

Survival of HIV patients was changed dramatically with the introduction of ART in Cape Verde at the end of 2004. The number of adults and children receiving ART has increased to 996–933 adults and 63 children in 2013—and the mortality rate among HIV patients receiving ART fell from 7.0% in 2009 to 4.1% in 2011 [6]. Currently ART is free and is available in 22 centres spread across the country’s islands. ART is initiated whenever CD4 count is lower than 350 cells/mm^3^. After testing positive, all samples are sent to the National Reference Laboratory (Laboratório ELISA in Hospital Dr. Agostinho Neto) for confirmation and CD4 count.

The island of Santiago accounts for the majority (73.8%) of new cases of HIV in Cape Verde, with the municipalities of Praia (capital) and Santa Catarina registering the most cases nationwide (Table 1). Currently Santiago has 14 centres that offer Voluntary Counselling and Testing and 13 ART centres.

There is evidence that early diagnosis and presentation to HIV care is important for the individual who lives with the infection as well as to control its spread in a population. Early diagnosis and treatment not only can improve the individual’s health condition but also reduce his/her infectivity, by decreasing the risk of transmission as a result of behaviour change [7,8,9].

However, many HIV patients still present late at clinics for care because: (a) people infected with HIV are not aware of their risk of infection and fail to seek testing; (b) even if aware of their risk, they face considerable barriers to access HIV testing; (c) HIV infected people may not seek or have access to medical care upon receiving their test results [10]. Thus, late presentation to HIV care can be attributed to late diagnosis, which includes barriers to access HIV testing, delays between diagnosis and care, including poor linkage between healthcare centres offering HIV testing and ART centres, or both [11].

There is still no consensus regarding the definition of delayed HIV diagnosis [10]. This study follows the definition of the UK Collaborative HIV Cohort (UK CHIC), which defines “late presentation” as one in which the CD4 cell count is below that when treatment should be initiated (currently CD4 cell count <350 cells/mL or AIDS) [12]. The European Late Presenter Group consensus statement adopted a similar definition in 2011 [13]. Although the CD4 count threshold of 350 cells/mm^3^ is being increasingly questioned as the biomedical justification for earlier initiation of treatment, this study follows the suggestion of MacCarthy *et al.* (2014) noting that “*the existing CD4 count threshold of 350 cells/mm^3^ should continue to be reported, even if other CD4 count thresholds are introduced to allow for more consistent comparisons across studies*” [14].

Despite the growing access to ART, a substantial proportion of HIV-infected individuals in high-income countries present at clinics for care with advanced or severe disease [15]. However, little is known in low- and middle-income countries, particularly in Sub-Saharan Africa, about the characteristics of HIV-patients who present late for care at clinics. Therefore, improving the knowledge about these HIV-patients in Sub-Saharan Africa may help to improve care as well as to design interventions to overcome existing barriers to early diagnosis and treatment.

The aim of this study is to examine the characteristics of people diagnosed with HIV on the island of Santiago, comparing patients who presented late to HIV care with those who presented earlier, and to explore factors associated with late presentation to HIV care, between 2004 and 2011.

## 2. Materials and Methods

### 2.1. Study Design

An unmatched case-control study was conducted in a population of patients diagnosed with HIV who used healthcare services on the island of Santiago to receive ART from 2004 to 2011. Patients with diagnosis of HIV infection prior to 2004—when ART was introduced in Cape Verde—were excluded from this analysis, leading to a final sample size of 368 patients.

### 2.2. Definition of Cases and Controls

Cases were all individuals aged 18 years or older, residents of the island of Santiago, diagnosed with HIV and receiving ART who had at first examination a CD4 lymphocyte count of less than 350 cells/mm^3^. The number of cases among the study sample was 191 patients. Controls were defined as all individuals aged 18 years or older, residents in Santiago, diagnosed with HIV and receiving ART who had at first examination a CD4 lymphocyte count of greater than or equal to 350 cells/mm^3^. The number of controls among the study sample was 177 patients.

### 2.3. Data Collection

Eligible participants (cases and controls) were identified though records at the ART centre and contacted by the research team for further data collection using a structured closed-end questionnaire applied in a face-to-face interview. Sociodemographic, socioeconomic, and behavioural characteristics of HIV patients were collected at the ART centre.

The dependent variable is defined as equal to one if the participant is a late presenter and zero otherwise. Independent variables collected in the questionnaire were the following: sociodemographic information such as gender, age (categorized as <30, 30–39, 40–49, 50–59 and ≥60), education level (none, basic, secondary, university), where basic corresponds to six years of education, type of job (informal economy, no job, others), job status (employed, unemployed), and marital status. Other factors include reason for HIV testing (personal reason and medical indication, only medical indication), status disclosure, and access conditions (“*easy*”, if the time of travel between home and health facilities is less than 30 min, and “*difficult*”, otherwise). At the health service level we considered the following information contained in the patient’s record: type of transmission (heterosexual, blood transfusion, unknown), and time to initiate ART after diagnosis (in months). The latter was computed using information contained in the patient’s record; diagnostic date is the one recorded in patient’s process; ART initiation is the date the patient initiated the treatment. As mentioned in the Introduction, in Cape Verde, it is recommended to initiate ART whenever CD4 count is lower than 350 cells/mm^3^. Other responses related with drug use, sexual behaviours, and stigma were not available.

### 2.4. Data Analysis

Data were inserted in a spreadsheet and exported into SPSS Statistics for Windows, Version 22.0 (IBM Corp., Armonk, NY, USA) to be analysed. Descriptive statistics were conducted for sociodemographic and behavioural characteristics of study participants. Both bivariate and multivariate logistic regression models were used to study the association between each independent variable and the outcome of interest: late presentation to HIV care. Factors statistically significant at the 20% level (*p*-value < 0.20) in the bivariate analysis remained in the multivariate model to control for the effect of confounders. The magnitudes of associations were estimated by means of Unadjusted and Adjusted Odds Ratios (UAR and AOR) with 95% confidence intervals.

### 2.5. Ethics Approval

This study was approved by the National Ethics Committee for Health Research (Cape Verde) in April 2012. To ensure confidentiality, names were not mentioned in either the questionnaire or in the database, and consent was obtained prior to data collection. The Ethics approval number is No 11/2012, 26 April 2012.

## 3. Results

### 3.1. Study Population

Among the 368 subjects (191 cases and 177 controls) included in the study, 51.9% had a CD4 lower than 350 cells/mm^3^ at ART initiation. One hundred and ten (57.7%) cases and 105 (59.3%) controls were female. The median age was 44 years for cases and 43 years for controls. The majority of study cases (34.5%) and controls (29.4%) were in the age group of 40–49 years. Those with basic education (six years in Cape Verde), single, and employed dominated both study groups (Table 2).

Three hundred and five (91.0%) patients did not disclose HIV status to their family and friends, of which 176 (52.5%) were cases and 159 (47.5%) were controls (Table 2). Amongst the study subjects, 240 (65.2%) tested for HIV by medical indication, of whom 157 (65.4%) were cases and 83 (34.6%) were controls. Median length between HIV diagnosis and ART initiation was 30 days (2 months) for cases and 360 days (18 months) for controls, in which 40.3% of cases presented for ART less than 30 days after the first CD4 count due to serious immunologic condition (Table 2).

### 3.2. Bivariate Analysis

Age and education were found to be associated with late presentation to HIV care based on bivariate analysis. Comparing with patients under 30, those in the age groups 30–39 (*p* = 0.07), 40–49 (*p* = 0.03), and older than 60 (*p* = 0.05) were more likely to present late to HIV care. Subjects with higher education were also more likely to present late to HIV care than those with no education (*p* = 0.04) (Table 2).

There is some evidence that marital status is associated with late presentation to care using bivariate analysis; patients separated were 1.8 times more likely to present late as compared to those married (*p* = 0.05). Reason for HIV testing and length between HIV diagnosis and ART initiation were also found to be associated with late presentation using bivariate analysis (Table 2).

### 3.3. Multivariate Analysis

First, multivariate analysis was conducted with all variables that were statistically significant (at the 20% level) in the bivariate analysis. Key variables like gender, age, and status disclosure were always included in the model, since they have shown to matter in peer-reviewed literature. The other variables retained are those that remain statistically significant at 5% level (*p* < 0.05). We did not include the variable length between HIV diagnosis and initiation of ART in the multivariate model because this is an endogenous variable (time to initiate ART is causing the outcome). Adjusting for other factors, individuals who undertook an HIV test by indication of their doctor were 4.8 times more likely to present late compared with those who tested for HIV on their own initiative (AOR = 4.84). Also, individuals aged 60 years or older were 3.19 times more likely to present late, when compared to those younger than 30 years (AOR = 3.19) (Table 3).

## 4. Discussion

Late presentation to HIV care is harmful to the patient and to society, and more costly [13]. In fact, late presentation for care increases the chances of deteriorating patients’ health status at the time of ART initiation, which plays an important role in the success of treatment [16]. Patients with advanced HIV disease at ART initiation are less likely to respond to treatment and have higher mortality rates compared with those who initiate ART earlier [17,18,19]. Also, as noted above, late presentation for care poses a higher cumulative risk of HIV transmission to others, while earlier presentation to ART can reduce viral load and, thus, the risk of transmission [15,20,21].

This is the first study in Cape Verde exploring factors associated with late presentation to HIV care. The main finding is that between 2004 and 2011, most patients on the island of Santiago were diagnosed with HIV following a medical appointment, and the majority (51.9%) are late presenters, *i.e.*, had a CD4 count below 350 cells/mm^3^—the level at which treatment is currently recommended in Cape Verde. For late presenters, the estimated average time between diagnosis and ART initiation is two months. This is a reasonable period meaning that delayed entry into treatment is not a major issue [22].

The figure found in our study for late presenters (51.9%) is lower than what is reported in studies in Nigeria and India, where authors reported 71.8% and 68.7% of those diagnosed with HIV infection with a CD4 cell count below 350 cells/mm^3^, respectively [23,24]. The current study found that patients who undertook an HIV test by indication of their doctor were more likely to present late to care than those who tested for HIV on their own initiative. Our findings also show that, in the bivariate model, earlier initiation of ART (following diagnosis) is associated with a higher probability of late presentation (AOR < 1), mainly because persons presenting with a CD4 count below 350 cells/mm^3^ are more likely to be put on treatment right away following national policies on treatment initiation. Similar results were obtained for a study on HIV positive persons in Europe [22].

In this research those in the age group 40–49 accounted for the largest proportion of people with late presentation (34.6%), and they were 2.2 times more likely to present late to HIV care than those aged <30 years. This fact may be partially explained by the higher incidence of HIV among people older than 40 years. Also, on average, older people may have become infected longer before their presentation to HIV care, and they tend to have a low self-perceived HIV risk, thus seeking an HIV test later than younger people, though this has not been shown in Africa [15,25,26]. Older age has been shown to compound the negative impact of late presentation on treatment outcomes in both Europe and North America [7], and Africa [27]. People aged 60 years and older accounted for 10.5% of participants with CD4 < 350 cells/mm^3^ and 7.9% with ≥350 cells/mm^3^. Those aged 60 years and older were 3.2 times more likely to present late to HIV care than those younger than 30 years. A possible explanation for late presentation among people in this age group could be the fact that health professionals tend to associate patients’ symptoms with the natural process of ageing and frequent conditions in this age group, postponing their concerns about HIV and other sexually transmitted infection (STIs) [28].

This study found that testing for HIV by medical indication increased the risk of late presentation. This finding is supported by other studies [18,29], which highlight that timely access to healthcare is not the only factor playing a role in early HIV presentation since other factors such as being unaware of HIV infection, risk behaviour, and not testing for HIV are associated with late presentation.

In this study, 92.1% of cases and 89.8% of controls did not disclose HIV status to their family and friends. This finding is supported by previous research conducted in Africa. A study conducted in Ethiopia (Southern Tigray Zone) found a lower proportion of non-disclosure both among cases (64.4%) and controls (41.0%) [30]. That study also found that patients who did not disclose their status to their partner were more likely to present late to HIV care [30]. Qualitative findings from another case-control study in Ethiopia (Southwest), also showed that non-disclosure of HIV status was a major barrier for late presentation for HIV/AIDS care [10]. Other studies conducted in Uganda [16] and Kenya [31] revealed that HIV status disclosure was associated with timely HIV/AIDS care. The literature suggests that hiding HIV-positive status may delay or inhibit presentation to HIV care. Other studies have shown that the rates of sero-status disclosure among sexual partners in Africa are low [32,33].

The major limitations of this study are its design and selection bias. Only patients diagnosed with HIV who used healthcare services in the island of Santiago to receive ART were included in this study. Thus, this analysis of late presentation does not represent patients infected with HIV on the island of Santiago who did not attend the clinic. Nevertheless, our study analysed, for the first estimate, late presentation and factors associated with it in an African country underrepresented in HIV research publications. The questionnaire used did not assemble information regarding perceived or experienced HIV stigma, which may be one important reason for delaying HIV testing and counselling in Sub-Saharan Africa [34]. Another limitation is related to estimation methods. In order to address the endogeneity problem more sophisticated approaches, such as instrumental variables or General Method of Moments (GMM) should be considered.

This study includes a considerable number of HIV-patients for a country like Cape Verde, providing demographic, psychosocial, and behavioural characteristics of patients who presented late to HIV care in that country. The major implication of the findings from this study is that more effort should be made to early diagnose HIV infection in Cape Verde. Delayed testing has an impact on the wellbeing of patients and on the future spread of the epidemic among populations and groups.

## 5. Conclusions

This study found that HIV positive individuals who undertook an HIV test by indication of their doctor were more likely to present late than those who tested for HIV on their own initiative. Also, the shorter was the length of time between HIV diagnosis and ART initiation, the more likely subjects were to have been late presenters. Results from this study suggest that low CD4 count at ART initiation might be due to high frequency of late presentation amongst new HIV-positive patients, as observed in other studies in Sub-Saharan Africa. Late presentation is an important obstacle to the success of large-scale ART.

This study shows that routine HIV testing and counselling should be strengthened in Cape Verde, targeting people aged 60 years and older. Further research is needed in order to better understand reasons for late presentation for HIV care. Also, future research should address the role of stigma and discrimination in the decision of testing for HIV in Cape Verde.

## Figures and Tables

**Table 1:** New cases of HIV infection diagnosed in Cape Verde, by Health delegacy, 2012.

Municipalities	Male *n* (%)	Female *n* (%)	Unknown *n* (%)	Total *n* (%)
Praia	76 (43.7)	96 (55.2)	2 (1.1)	174 (49.6)
Santa Catarina	27 (50.0)	27 (50.0)	0 (0.0)	54 (15.4)
São Vicente	16 (53.3)	14 (46.7)	0 (0.0)	30 (8.5)
Boavista	8 (38.1)	13 (61.9)	0 (0.0)	21 (6.0)
São Filipe	10 (50.0)	10 (50.0)	0 (0.0)	20 (5.7)
Santa Cruz	7 (58.3)	5 (41.7)	0 (0.0)	12 (3.4)
Sal	6 (60.0)	4 (40.0)	0 (0.0)	10 (2.8)
Tarrafal	1 (12.5)	7 (87.5)	0 (0.0)	8 (2.3)
São Miguel	4 (66.7)	2 (33.3)	0 (0.0)	6 (1.7)
Brava	0 (0.0)	4 (100.0)	0 (0.0)	4 (1.1)
São Domingos	1 (33.3)	2 (66.7)	0 (0.0)	3 (0.9)
Maio	2 (66.7)	0 (0.0)	1 (33.3)	3 (0.9)
Ribeira Grande	1 (50.0)	1 (50.0)	0 (0.0)	2 (0.6)
Mosteiros	1 (50.0)	1 (50.0)	0 (0.0)	2 (0.6)
Paúl	0 (0.0)	1 (100.0)	0 (0.0)	1 (0.3)
São Nicolau	1 (100.0)	0 (0.0)	0 (0.0)	1 (0.3)
Porto Novo	0 (0.0)	0 (0.0)	0 (0.0)	0 (0.0)
Total (%)	161 (45.9)	187 (53.3)	3 (0.9)	351 (100.0)

Source: Ministry of Health [6].

**Table 2:** Sociodemographic characteristics of study participants and factors associated with late presentation to HIV care (CD4 < 350 cells/mm^3^), Santiago island (Cape Verde), 2011.

User Characteristics	Total *n* (%)	Cases CD4 < 350 *n* (%)	Controls CD4 ≥ 350 *n* (%)	UOR (95% CI)	*p*-Value
**Median CD4 Count (Min., Max.)**		113 (2, 345)	466 (350, 1112)		
**Gender**					
Male	**153 (41.6)**	81 (42.4)	72 (40.7)	1 (Reference)	
Female	**215 (58.4)**	110 (57.6)	105 (59.3)	1.07 (0.72–1.65)	0.73
**Age group**					
<30	**41 (11.1)**	15 (7.9)	26 (14.7)	1 (Reference)	
30–39	**99 (26.9)**	53 (27.7)	46 (26.0)	2.00 (1.21–4.21)	0.07
40–49	**118 (32.1)**	66 (34.5)	52 (29.4)	2.20 (1.25–4.29)	0.03 *
50–59	**76 (20.7)**	37 (19.4)	39 (22.0)	1.64 (0.95–2.62)	0.21
≥60	**34 (9.2)**	20 (10.5)	14 (7.9)	2.47 (1.33–4.45)	0.05
**Education**					
None	**94 (25.5)**	47 (24.6)	47 (26.5)	1 (Reference)	
Basic	**171 (46.5)**	98 (51.3)	73 (41.2)	0.66 (0.39–1.09)	0.10
Secondary	**94 (25.5)**	41 (21.5)	53 (29.9)	1.36 (0.76–2.44)	0.32
University	**9 (2.5)**	5 (2.6)	4 (2.3)	1.75 (0.18–3.13)	0.04 *
**Access**					
Easy	**256 (69.6)**	130 (68.1)	126 (71.2)	1 (Reference)	
Difficult	**112 (30.4)**	61 (31.9)	51 (28.8)	1.00 (0.63–1.52)	0.21
**Job**					
Others	**192 (52.2)**	101 (52.9)	91 (51.4)	1 (Reference)	
Informal economy	**33 (9.0)**	17 (8.9)	16 (9.0)	1.03 (0.49–2.17)	0.92
No job	**143 (38.8)**	73 (38.2)	70 (39.6)	1.02 (0.65–1.56)	0.95
**Marital status**					
Married	**52 (14.1)**	21 (11.0)	31 (17.5)	1 (Reference)	
Single	**201 (54.6)**	111 (58.1)	90 (50.9)	1.43 (0.83–2.63)	0.31
Widow	**58 (15.8)**	19 (9.9)	39 (22.0)	0.00 (0.01–0.05)	0.99
Separated	**14 (3.8)**	2 (1.1)	12 (6.8)	1.82 (0.92–2.81)	0.05 *
Cohabiting	**43 (11.7)**	38 (19.9)	5 (2.8)	2.33 (1.27–4.31)	0.06
**Job status**					
Unemployed	**133 (36.1)**	68 (35.6)	65 (36.7)	1 (Reference)	
Employed	**235 (63.9)**	123 (64.4)	112 (63.3)	1.00 (0.65–1.53)	0.99
**Transmission**					
Heterosexual	**335 (91.0)**	176 (92.1)	159 (89.8)	1 (Reference)	
Blood transfusion	**10 (2.7)**	4 (2.1)	6 (3.4)	1.09 (0.31–3.85)	0.88
Unknown	**23 (6.3)**	11 (5.8)	12 (6.8)	1.01 (0.46–2.22)	0.96
**Reason for HIV test**					
Personal/both	**128 (34.8)**	34 (17.8)	94 (53.1)	1 (Reference)	
Medical indication	**240 (65.2)**	157 (82.2)	83 (46.9)	5.23 (2.59–7.91)	<0.01 *
**Status disclosure**					
No	**335 (91.0)**	176 (92.1)	159 (89.8)	1 (Reference)	
Yes	**33 (9.0)**	15 (7.9)	18 (10.2)	1.10 (0.54–2.22)	0.79
**Length between HIV diagnosis and initiation of ART (average in months)**	**10 (100.0)**	2 (51.9)	18 (48.1)	0.94 (0.65–1.19)	<0.01 *

* Statistically significant at 5% level.

**Table 3:** Multivariate results (unadjusted and adjusted Odds Ratio) for variables associated with late presentation to HIV care (CD4 < 350 cells/mm^3^), Santiago island (Cape Verde), 2011.

User Characteristics	Bivariate		Multivariate Model	
	UOR (95% CI)	*p*-Value	AOR (95%CI)	*p*-Value
**Gender**				
Male	1 (Reference)		1 (Reference)	
Female	1.07 (0.72–1.65	0.73	1.00 (0.64–1.58)	0.10
**Age group**				
<30	1 (Reference)		1 (Reference)	
30–39	2.00 (1.21–4.21)	0.07	2.06 (0.93–4.57)	0.08
40–49	2.20 (1.25–4.29)	0.03 *	2.20 (1.01–4.81)	0.05
50–59	1.64 (0.95–2.62)	0.21	1.71 (0.74–3.94)	0.21
≥60	2.47 (1.33–4.45)	0.05 *	3.19 (1.16–8.78)	0.03 *
**Reason for HIV test**				
Personal/both	1 (Reference)		1 (Reference)	
Medical indication	5.23 (2.59–7.91)	<0.01 *	4.84 (2.99–7.84)	<0.01 *
**Status disclosure**				
No	1 (Reference)		1 (Reference)	
Yes	1.10 (0.54–2.22)	0.79	0.77 (0.36–1.64)	0.50

* Statistically significant at 5% level.

## Abbreviations

The following abbreviations are used in this manuscript:

95%CI	95% Confidence Interval
AIDS	Acquired Immunodeficiency Syndrome
AOR	Adjusted Odds Ratio
ART	Antiretroviral therapy
HIV	Human Immunodeficiency Virus
STI	Sexually Transmitted Infections
UOR	Unadjusted Odds Ratio
